# Shedding Light on the Aftermath: Childhood Maltreatment’s Role in Modifying the Association Between Recent Life Stress and Resting-State Network Connectivity

**DOI:** 10.3390/bs14100958

**Published:** 2024-10-16

**Authors:** Jingjing Luo, Jianjun Zhu, The NSPN Consortium, Yuanyuan Chen

**Affiliations:** 1 Center for Early Environment and Brain Development, School of Education, Guangzhou University, Guangzhou 510006, China; 2 Department of Psychology, Guangzhou University, Guangzhou 510006, China; 3 Department of Special Education, Guangzhou University, Guangzhou 510006, China

**Keywords:** childhood maltreatment, recent life stress, resting-state network connectivity, longitudinal study, moderation effect

## Abstract

Childhood maltreatment has been demonstrated to impact brain development. However, whether childhood maltreatment can influence the effects of recent stress on brain networks remains unclear. This study aimed to investigate whether childhood maltreatment moderates the longitudinal relationship between recent life stress and within- and between-network connectivity in key brain networks, including the anterior salience (ASN), central executive (CEN), default mode (DMN), and emotional regulation network (ERN). A cohort of 172 individuals from the Neuroscience in Psychiatry Network (NSPN) underwent MRI scans at two specific time points and undertook evaluations of childhood maltreatment and recent life stress. The results showed that childhood abuse moderated the association of recent life stress with the within-network connectivity of ASN and ERN but not DMN and CEN. Furthermore, recent life stress significantly interacted with childhood abuse to be associated with the between-network connectivity of ASN-DMN, ASN-CEN, ASN-ERN, DMN-ERN and CEN-ERN. Overall, among youth exposed to higher degrees of childhood abuse, greater recent life stress was longitudinally associated with increased network connectivity. Understanding these interactions can provide valuable insights for developing prevention strategies and interventions aimed at mitigating the lasting impact of childhood maltreatment on brain development and overall well-being.

## 1. Introduction

Childhood maltreatment is highly prevalent and affects more than one third of the adult population [1]. This form of trauma contributes significantly to the development of various psychiatric disorders with prevalence estimates ranging from 30% to 64% [2,3,4]. Extensive research has elucidated that maltreatment can precipitate profound alterations in the functional connectivity of the brain’s principal resting-state networks (RSNs) [5,6,7]. Resting-state networks represent specific patterns of brain activity that emerge when an individual is not focused on any particular, goal-oriented tasks [8]. Empirical evidence suggests that maltreatment is associated with significant changes in neural connectivity within key RSNs implicated in threat processing, salience detection, and higher-order cognitive functions [9,10,11]. Specifically, threat processing refers to the brain’s mechanisms for learning about and rapidly responding to threats [12], salience detection delineates the brain’s ability to identify and react to novel information and external stimuli [13], and higher-order cognition involves advanced mental processes such as concept formation and strategic decision making. These RSNs play a pivotal role in daily functioning and overall well-being. However, the potential role of childhood abuse or neglect as a risk factor influencing the association between recent life stress and changes in subsequent key resting-state network connectivity remains unexplored. By investigating this unexplored area, our study aims to deepen the understanding of how childhood maltreatment shapes neural responses to current stressors, aiming to improve intervention strategies and theoretical models in mental health and neurodevelopmental research. This investigation is crucial for understanding the complex relationship between childhood trauma, present stress, and neural adaptation, contributing to both academic understanding and practical approaches in addressing the long-term effects of childhood maltreatment.

The Triple Network Model of mental disorders has significantly contributed to our understanding of psychiatric disorders, particularly regarding the crucial roles played by three key networks: the anterior salience network (ASN), central executive network (CEN), and default mode network (DMN) [14,15]. The ASN links the anterior insula with the dorsal anterior cingulate cortex and plays a key role in detecting saliency and capturing attention [16,17]. The DMN, rooted in the posterior cingulate cortex and medial prefrontal cortex, is associated with self-referential thinking, emotional processing, and thoughts that are independent of external stimuli [18,19]. The CEN, including regions of the dorsolateral prefrontal cortex and posterior parietal cortex, is fundamental for cognitive control and working memory functions [16,20]. An additional network of interest is the emotional regulation network (ERN) [21], which encompasses the inferior frontal gyrus, middle temporal gyrus, and precentral gyrus. The ERN is crucial for regulating emotions cognitively and managing responses to stress, mainly by influencing limbic circuits such as the amygdala [22,23]. This network interacts dynamically with the ASN in detecting and prioritizing emotional stimuli [24,25], with the CEN in exerting cognitive control over emotional responses [20,26], and with the DMN in maintaining self-referential emotional processes [27,28]. Understanding how these networks integrate is essential for a comprehensive view of brain function in both typical and pathological contexts.

Prior evidence has linked recent stressful events with changes in network connectivity. Stressful events have been associated with decreased functional connectivity between the DMN and ASN [29,30] and increased connectivity within the cortico-limbic network [31]. Some studies examining the controlled manipulation of acute stress have found that psychosocial stress is associated with decreased whole-brain network efficiency [32], increased connectivity between the DMN and CEN regions [29], and decreased connectivity between the ASN and DMN/CEN [29,33]. Notably, healthy individuals have been shown to have increased functional connectivity within the ASN under acute stress [34], while individuals with PTSD or a history of stress exhibit reduced connectivity within the ASN and DMN as well as altered synchronization between the DMN and CEN [35]. Recent studies have also highlighted the role of the emotional regulation network (ERN) in stress responses, showing that acute stress can lead to increased connectivity within the ERN [36,37], which is crucial for adaptive emotional regulation and maintaining psychological stability [36,38]. However, prolonged or intense stress may disrupt ERN connectivity, impairing its ability to modulate limbic activity and leading to maladaptive emotional responses [39]. These mixed findings suggest that stress has varying effects on neural networks and led us to investigate whether childhood maltreatment modified the association between recent stress and network connectivity.

The stress-sensitization model proposes that experiencing childhood maltreatment could make individuals more susceptible to mental health risks in response to stressors that occur later in life [40]. Empirical evidence supports this hypothesis, showing that individuals who experience childhood maltreatment are more prone to major depression, PTSD, anxiety disorders, and transdiagnostic psychopathology after recent stressful events than those who were not exposed to maltreatment during their childhood [41,42]. Although the stress sensitization model is a fundamental concept in mental health research, it remains unclear whether this model can explain the interaction between recent stressors and childhood maltreatment in large-scale resting-state networks. Two studies have shown that this interaction can modulate orbitofrontal gray matter volume and seed-based connectivity involved in emotional processing [43,44]. However, exploring large-scale resting-state network connectivity provides a more comprehensive understanding of brain function, and it has the potential to capture global changes in brain connectivity outside specific regions of interest.

To address this gap in the literature, we conducted a longitudinal study to investigate the potential interaction between childhood maltreatment and recent stressful events in four networks: the ASN, DMN, CEN, and ERN. Consistent with the stress sensitization model, our hypothesis posits that childhood maltreatment would amplify the longitudinal effects of recent stressful life events on both within- and between-network connectivity. Specifically, we hypothesize that (1) childhood maltreatment will intensify the longitudinal impact of recent stressful life events on within-network connectivity; and (2) childhood maltreatment will similarly enhance the longitudinal influence of recent stressful life events on between-network connectivity.

This study offers a novel perspective on the neural mechanisms underlying differential stress sensitivity by examining how childhood maltreatment moderates the impact of recent life stress on large-scale brain network connectivity. Unlike previous research that has primarily focused on specific brain regions, our approach provides a comprehensive view of how early-life adversity influences neural adaptation to stress across development. Elucidating these moderating effects is crucial for advancing theories of neurodevelopment and psychopathology, particularly in understanding individual differences in stress reactivity and resilience. Clinically, our findings may facilitate the identification of neural biomarkers that indicate heightened vulnerability to mental health disorders in individuals with a history of childhood maltreatment. These biomarkers could be pivotal in developing targeted interventions and prevention strategies aimed at enhancing resilience and reducing long-term psychological consequences. Additionally, understanding these neural dynamics could support the development of personalized therapeutic approaches that integrate both early-life adversity and recent stressors, ultimately improving mental health outcomes. By bridging a critical gap in the literature, this study lays the groundwork for future research to explore the complex interactions between early adverse experiences and current life stressors in shaping mental health trajectories.

## 2. Materials and Methods

### 2.1. Participants

A total of 172 individuals were selected from the Neuroscience in Psychiatry Network (NSPN) cohort, which tracks 2406 participants aged between 14 and 24, living in regions of north London and Cambridgeshire, UK, as part of a longitudinal study. Data were collected between March 2013 and September 2019. Throughout the study, participants completed Home Questionnaire Packs (HQPs) and sociodemographic questionnaires at three different intervals (HQP1, HQP2, and HQP3). Additionally, they underwent two in-unit assessments (IUA1 and IUA2), including cognitive assessments, magnetic resonance imaging (MRI), and questionnaires.

For this specific study, 172 participants underwent fMRI scans at two time points (IUA1 and IUA2) to obtain resting-state brain network connectivity data. Childhood maltreatment was measured at IUA1, and recent life stress was measured at least two months before IUA2. Participants were excluded if they had a history of receiving psychiatric treatment, neurological conditions, head trauma, or any form of intellectual impairment. Written informed consent was obtained from participants aged 16–24, while those aged 14–15 required written consent from a legal guardian in addition to their own assent. The NSPN project received approval from the Cambridge Central Research Ethics Committee, while the protocol for secondary data analysis was sanctioned by the Institutional Review Board of our university.

Basic information about the participants in this study is detailed in Table 1. To ensure the statistical robustness of our findings, we conducted a power analysis for a multiple regression. Based on the analysis, a sample size allowing approximately 64 degrees of freedom for error was needed to detect an effect size of 0.15 with 80% statistical power at a 0.05 significance level. With 172 participants, our study comfortably exceeds this threshold, confirming the adequacy of our sample size for detecting the intended effects. The recruitment utilized an accelerated longitudinal design within the NSPN to capture developmental trajectories over a broad age range. Multifaceted recruitment strategies included leveraging school networks, community outreach, and public health records to assemble a representative sample. Compensation was provided to participants based on the time and effort required for each component of the study. Detailed descriptions of the recruitment process and the overall sample composition can be found in the Supplementary Materials.

### 2.2. Measures

#### 2.2.1. Childhood Maltreatment

Childhood maltreatment was assessed at IUA1 using the Childhood Trauma Questionnaire (CTQ), which is a comprehensive 28-item retrospective self-report questionnaire designed to evaluate five sub-dimensions of maltreatment: emotional and physical abuse, sexual abuse, as well as emotional and physical neglect [45]. Responses on the CTQ are rated on a five-point Likert scale, ranging from 1 (‘never true’) to 5 (‘frequently true’). To quantify childhood abuse, the scores of items pertaining to emotional, physical, and sexual abuse (15 items in total) were added. Similarly, the scores of items related to emotional and physical neglect (10 items in total) were added to represent childhood neglect. Greater exposure to childhood abuse or neglect was reflected by higher scores on these scales. We conducted a confirmatory factor analysis on the items of the CTQ, and the model fit was found to be acceptable (χ^2^/df = 1.96, CFI = 0.91, RMSEA = 0.076, TLI = 0.885). This indicates the reasonable structural validity of the questionnaire in our study. The measures demonstrated good internal consistency in this sample with a Cronbach’s alpha of 0.79 for items measuring abuse and 0.82 for items measuring neglect.

#### 2.2.2. Recent Stressful Events

Recent stressful events were assessed at HQP2 using the Life Events Questionnaire (LEQ) [46], which is a self-report instrument designed to capture significant life events experienced by individuals within the previous 18 months. For this sample, the LEQ demonstrated a Cronbach’s alpha coefficient of 0.76, which is indicative of satisfactory internal consistency. This includes a variety of significant life events, such as changing schools, jobs, or colleges; alterations in family structure; moving to a new home; household disasters like fires, floods, or burglaries; serious illnesses affecting the participant or close ones; hospital stays; deaths; the loss of a family pet; losing contact with friends; or difficulties in friendships. In cases where multiple events occurred within each domain, participants were asked to reflect on the most unpleasant, sad, or painful event and provide additional information. They were then required to rate the primary event on a scale ranging from very pleasant, pleasant, neither, quite unpleasant, or very unpleasant. Furthermore, participants indicated whether the event impacted them for two weeks or more. Events rated as quite unpleasant or very unpleasant that had a lasting impact of two weeks or more were considered to have a more significantly distressing effect on individuals. Consequently, these events were scored, and this variable, representing these specific events, served as the primary life-event variable in most analyses.

### 2.3. Magnetic Resonance Imaging Acquisition and Pre-Processing

Functional MRI (fMRI) was performed at two research locations using identical 3T MRI systems (Magnetom TIM Trio; Siemens Healthcare, VB17 software). A multi-parametric mapping (MPM) protocol was employed to obtain high-resolution T1-weighted images for anatomical alignment and ROI localization. The T1-weighted images were acquired using the following parameters: repetition time (TR) = 18.7 ms, echo times (TE) = 6 equidistant acquisitions between 2.2 and 14.7 ms, flip angle (α) = 20°, voxel resolution = 1 mm^3^, field of view (FOV) = 256 × 240 mm, 176 sagittal slices, and readout bandwidth = 425 Hz/pixel. During the MRI acquisition, participants were instructed to remain still, keep their eyes closed, and relax to minimize head motion. Additionally, all participants used a headrest and fixation devices to further reduce motion artifacts.

Resting-state fMRI data were collected over a 10-minute period, which is a duration chosen based on prior studies demonstrating that this length of time is sufficient to capture reliable and stable resting-state functional connectivity patterns [47,48]. The multi-echo echo-planar imaging (EPI) sequence consisted of 263 volumes with the following parameters: repetition time (TR) = 2.42 s; GeneRalized Autocalibrating Partial Parallel Acquisition (GRAPPA) factor of 2; matrix size = 64 × 64 × 34; field of view (FOV) = 240 × 240 mm; in-plane resolution = 3.75 × 3.75 mm; slice thickness = 3.75 mm with a 10% inter-slice gap; bandwidth = 2368 Hz/pixel; and echo times (TE) = 13, 30.55, and 48.1 ms.

The preprocessing of functional MRI data involved correcting for slice timing, adjusting for motion, and applying spatial normalization. First, functional images were coregistered to the T1-weighted structural images to ensure precise alignment between the functional and anatomical data. The transformation matrix derived from the normalization of the T1 images was then applied to the functional data, enabling accurate network localization within the standard brain template. Further details on data preprocessing strategies are provided in the eMethods section, ‘Image Preprocessing,’ in the Supplementary Materials.

### 2.4. Functional Connectivity Analyses

We selected the ERN regions of interest (ROIs) a priori based on a meta-analysis [21] utilizing activation likelihood estimation. This meta-analysis, which encompassed 23 studies with 479 overall participants, identified a comprehensive emotion regulation network. ROIs for the CEN and ASN were obtained from a publicly available resting-state network atlas derived from independent component analysis [49]. DMN ROIs were determined through a meta-analysis of its connectivity patterns [50]. For each node within the networks, a 5 mm sphere was centered at the coordinates corresponding to the peak activation of each distinct cluster.

**Figure 1:**
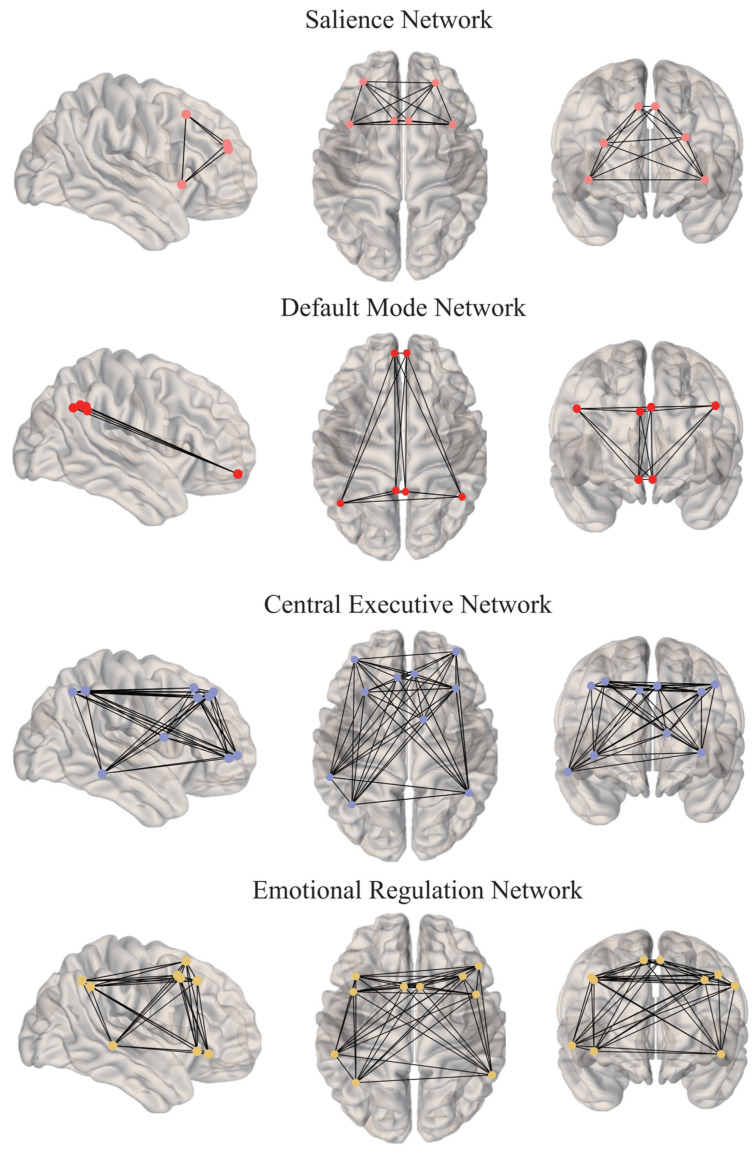
Axial, coronal and sagittal slices displaying the regions of interest within the resting-state functional connectivity networks.

Three-dimensional brain images were generated using the brainconn package in R, mapping the ROIs onto the MNI152 standard brain template. A 5 mm sphere was created around each peak activation coordinate, representing the functional nodes of interest within each network, as shown in Figure 1. The BOLD time series for each ROI was defined as the average time course of the voxels within the sphere. Within-network functional connectivity was quantified as the average of Pearson’s correlation coefficients computed for all pairs of Fisher’s z-transformed ROIs within each network. In contrast, connectivity between networks was determined by averaging the correlations for ROI pairs across different networks. As outlined in previous studies [51,52], the confounding influence of head movement on connectivity, following ME-ICA preprocessing, was addressed by regressing both within- and between-network connectivity against the mean framewise displacement (FD).

### 2.5. Data Analysis

Analyses were conducted in the R statistical software (version 4.0.2, R Project for Statistical Computing) from June 2022 to March 2023. First, we employed multiple regression analyses to examine the relationship between recent life stress at HQP2 and within- or between-network connectivity at IUA2. In these models, we adjusted for confounders such as gender, age, mean frame displacement (FD), and network connectivity at baseline (IUA1). This adjustment helps to account for any preexisting differences in connectivity that could influence our results.

Next, we investigated how maltreatment scores at IUA1 moderated the relationship between recent life stress measured at HQP2 and network connectivity assessed at IUA2. Multiple regression analyses were conducted, including main effects for recent life stress and childhood maltreatment and the interaction of recent life stress and childhood maltreatment on network connectivity. We examined the moderating influences of childhood abuse and neglect separately. Simple slope tests investigated the nature of significant interaction effects by determining whether the relationship between recent life stress and network connectivity differed significantly from zero at 1 SD above and 1 SD below the average childhood maltreatment score.

In this study, we employed the false discovery rate (FDR) correction method proposed by Benjamini and Hochberg [53] to manage the issue of multiple comparisons. Specifically, the FDR was controlled at a 0.05 threshold across all within- and between-network analyses included in the regression models. A small proportion (0–1%) of participants had missing data for childhood maltreatment and recent stressful events, and these missing data were handled using full-information maximum-likelihood estimation. Given the minimal amount of missing data, this method was considered more suitable than excluding participants, as exclusion could have compromised the representativeness of the sample [54]. No outliers were identified in the data, with values falling within 3 times the interquartile range from the 25th and 75th percentiles, which ensured the robustness of the regression analyses.

To further investigate potential age- and gender-related differences in functional connectivity, we divided participants into two age groups: the younger age group (the first two age bins) and the older age group (the last three age bins). Independent samples t-tests were conducted to compare the average connectivity strength within and between networks (ERN, CEN, DMN, ASN) across these two age groups. The same analytical approach was applied to assess gender-related differences in functional connectivity by comparing the connectivity strength within and between networks across male and female participants.

## 3. Results

### 3.1. Descriptive Analyses

As shown in Table 1, the study included data from 172 individuals with usable data (85 female [49.4%]; mean [SD] age, 18.7 [2.8] years; age range, 14–24 years). The mean score for childhood abuse among these individuals was 18.15 (SD = 4.21), while the average score for childhood neglect was 14.10 (SD = 4.49). Additionally, the mean score reported for recent life stress was 4.26 (SD = 1.93). Pearson correlation coefficients were used for the associations between these variables with the results reported in Supplementary Table S3.

### 3.2. Association Between Recent Life Stress and Resting-State Network Connectivity

As shown in Table 2, the results of the multiple regression analyses indicated that no significant associations between recent life stress and within-network connectivity for the ASN (*β* = 0.110, *p*FDR = 0.477), DMN (*β* = −0.105, *p*FDR = 0.477), CEN (*β* = 0.086, *p*FDR = 0.518), or ERN (*β* = 0.055, *p*FDR = 0.607). Moreover, no significant associations were observed between the network connectivity of ASN-DMN (*β* = 0.073, *p*FDR = 0.518), ASN-CEN (*β* = 0.153, *p*FDR = 0.430), ASN-ERN (*β* = 0.078, *p*FDR = 0.518), DMN-CEN (*β* = 0.015, *p*FDR = 0.852), DMN-ERN (*β* = −0.048, *p*FDR = 0.612), and CEN-ERN (*β* = 0.119, *p*FDR = 0.477) when considering factors such as gender, age, head movement, and baseline network connectivity with FDR correction applied.

### 3.3. Moderating Influence of Childhood Maltreatment on the Association Between Recent Life Stress and Within-Network Connectivity

**Figure 2:**
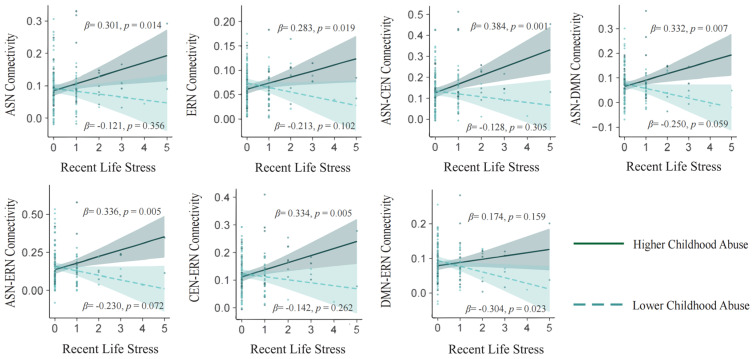
Within- and between-network connectivity as a function of recent life stress and childhood abuse. Note. Regression lines, dashed for low levels (1 SD below the mean) and solid for high levels (1 SD above the mean) of childhood abuse, are presented. Gender, age, and IUA1 network connectivity were incorporated as covariates in the moderation analyses. Abbreviations: ASN—anterior salience network, DMN—default mode network, CEN—central executive network, ERN—emotional regulation network.

We conducted separate analyses to examine the moderating influence of childhood abuse and neglect. The results revealed that childhood abuse moderated the association between recent life stress and within-network connectivity of the ERN (*β* = 0.245, *p*FDR = 0.028) and ASN (*β* = 0.209, *p*FDR = 0.050) but not between the DMN (*β* = 0.143, *p*FDR = 0.162) and CEN (*β* = 0.153, *p*FDR = 0.134). This moderating influence was observed after controlling for gender, age, head movement, and network connectivity at baseline (Table 3). Figure 2 illustrates these findings through a simple slope decomposition of the interaction effect. Specifically, in individuals with higher exposure to childhood abuse, a stronger longitudinal association was observed between increased recent life stress and the within-network connectivity of the ERN and ASN. These results remained significant even after including socioeconomic status and education level as covariates. Detailed results are provided in Supplementary Table S6.

### 3.4. Moderating Influence of Childhood Maltreatment on the Association Between Recent Life Stress and Between-Network Connectivity

Recent life stress significantly interacted with childhood abuse and, after adjusting for covariates, showed between-network associations of ASN-DMN (*β* = 0.288, *p*FDR = 0.021), ASN-CEN (*β* = 0.253, *p*FDR = 0.025), ASN-ERN (*β* = 0.280, *p*FDR = 0.021), DMN-ERN (*β* = 0.237, *p*FDR = 0.031), and CEN-ERN (*β* = 0.236, *p*FDR = 0.028) but not CEN-DMN (*β* = 0.207, *p*FDR = 0.057) (Table 3). As shown in Figure 2, greater recent life stress was longitudinally associated with increased ASN-DMN, ASN-CEN, ASN-ERN, and CEN-ERN connectivity in participants exposed to higher degrees of childhood abuse. However, participants who experienced lower degrees of childhood abuse demonstrated decreased DMN-ERN connectivity corresponding with increased recent life stress. These findings also remained robust after adjusting for socioeconomic status and education level, as detailed in Supplementary Table S6.

Moreover, when considering the covariates, we found that childhood neglect did not serve as a moderating factor in the relationship between recent life stress and within- or between-network connectivity. Detailed results are reported in Supplementary Table S2.

### 3.5. Analysis of Age and Gender Differences

No significant differences were found in within-network or between-network connectivity measures between the younger and older age groups (all *p* > 0.05), indicating that the functional connectivity patterns remain stable across the age range of our sample. Detailed results are provided in Supplementary Table S4. Similarly, no significant gender differences were observed in within-network or between-network connectivity measures (all *p* > 0.05). These findings suggest that in this sample, the functional connectivity strength is not significantly influenced by either age or gender. Comprehensive results are presented in Supplementary Table S5.

## 4. Discussion

This cohort study explored how childhood maltreatment influences the longitudinal relationship between recent life stress and resting-state network connectivity in individuals aged 14 to 24. The results indicate that childhood maltreatment could serve as a risk factor, potentially altering the impact of recent stress on subsequent neural connectivity. Specifically, individuals who reported higher degrees of childhood abuse showed a positive association between recent life stress and within-network connectivity of the ASN and ERN as well as the between- network connectivity of the ASN-DMN, ASN-CEN, ASN-ERN, DMN-ERN, and CEN-ERN several months later. By contrast, these associations were not significant among individuals who reported lower degrees of childhood abuse.

Our findings support the stress sensitization model proposed by Hammen et al. [40]. Specifically, childhood abuse increased the susceptibility of neural network connectivity to recent life stress. This is consistent with previous research showing that individuals who have experienced childhood maltreatment are at greater risk of developing major depression, PTSD, anxiety disorders, and other transdiagnostic psychopathologies following exposure to recent stressors [41,42]. However, this study was the first attempt to extend the stress sensitization model to include abnormal changes in neural network development. Additionally, empirical evidence suggests that the severity of childhood maltreatment correlates with distinctive cortisol response patterns during stress, providing a physiological foundation for the neurodevelopmental changes observed. These alterations in cortisol levels suggest a dysregulation of the hypothalamic–pituitary–adrenal (HPA) axis, indicative of a biological embedding of early adverse experiences, which likely underpins the changes in network connectivity detected in our MRI studies [55]. These findings collectively underscore the critical importance of early interventions targeted at children who have experienced abuse, which are aimed at mitigating neural changes induced by later stressful stimuli and prevent the onset of psychopathologies.

By measuring childhood maltreatment at IUA1 and recent life stress prior to IUA2, and observing changes in brain connectivity at IUA2, the study could more confidently attribute observed neural changes to the interaction of these factors over time. Our findings support this methodological approach: we observed that the interaction effect between childhood abuse and recent life stress was associated with the within-network connectivity of the ASN and ERN, supporting our first hypothesis. However, contrary to our anticipations, discernible impacts on the within-network connectivity of the DMN and CEN were not observed. These results suggest that the ASN and ERN may be more directly engaged in saliency detection and emotional regulation [17,23], which are processes that could have been affected by childhood abuse. Conversely, the DMN primarily engages in stimulus-independent thinking and attentional capture [19], which are functions that may not be directly influenced by the stressors linked to childhood abuse, potentially explaining the lack of significant interaction observed in our results. Similarly, the CEN, essential for executive functions like working memory and cognitive control [20], seems less impacted by childhood abuse and recent life stresses compared to networks directly involved in emotional processing and stress response, such as ASN and ERN.

In addition, our analysis demonstrated that parallel to within-network connectivity, the connectivity between the ASN and ERN, as well as the connectivity of these networks with other networks, were significantly influenced by the interaction between childhood abuse and recent life stress, thus supporting our second hypothesis. Notably, although the within-network connectivity of the DMN and CEN was not affected by the moderating effect, the network connectivity between the DMN/CEN and the other two networks was considerably impacted by the interplay between childhood abuse and recent life stress. This could be explained by the key role of the ERN and ASN in coordinating activity across the DMN and CEN to facilitate responses to threat stimuli and emotional regulation [10]. These results highlight the complex interplay between the different brain networks associated with cognitive functions, childhood abuse, and recent life stress. The observed modulation in connectivity underscores how childhood abuse fundamentally alters the brain’s response to new stressors. This indicates that both the development and subsequent functioning of the brain are modified in ways that change its response to external stimuli, reflecting a history of abuse.

Surprisingly, we discovered that childhood abuse, in conjunction with recent life stress, interacted in unexpected ways to predict the between-network connectivity of the DMN and ERN. Specifically, higher recent life stress was associated with decreased between-network connectivity in individuals who reported a lower degree of childhood abuse. Childhood abuse could have triggered hyperconnectivity between the DMN and the ERN as an adaptive response to future stressors. However, neural reliance on this hyperconnectivity may be disrupted by high degrees of recent life stress, leading to decreased between-network connectivity. In contrast, individuals with lower degrees of childhood abuse may not have developed this hyperconnectivity and thus may not be susceptible to such disruption after recent life stress. This finding underscores the importance of considering the heterogeneity of brain network development and its impact on the association between childhood abuse, recent life stress, and between-network connectivity.

We did not observe any significant association with childhood neglect. This lack of association may be attributed to two potential factors. First, the severity of neglect exposure in our sample may have been relatively low, limiting its capacity to produce detectable moderating effects on the relationship between recent life stress and network connectivity. Second, large-scale resting-state networks might be more responsive to the impacts of childhood abuse, while childhood neglect may instead heighten susceptibility to other neural markers, such as regional brain activation or seed-based connectivity, in response to recent life stress [43,44]. Future research should explore these differential effects further by employing more nuanced measures of neglect and assessing their long-term impact on brain development and stress reactivity. These hypotheses require additional investigation to fully understand the distinct neurobiological pathways through which childhood abuse and neglect influence stress-related brain network dynamics.

While our findings support the stress sensitization model, other theoretical frameworks could also provide valuable insights into the observed results. The cumulative risk model posits that multiple adversities together increase vulnerability to negative outcomes [56], while the diathesis-stress model suggests that inherent predispositions interact with stressors to influence individual responses [57]. In relation to our study, the cumulative risk model can explain the combined effect of childhood maltreatment and recent life stress on neural network changes, and the diathesis-stress model suggests that early neurobiological vulnerabilities from childhood abuse interact with later stress to alter network connectivity. Although our findings align most closely with the stress sensitization model, future research could benefit from integrating these alternative models to better understand the complex interactions between early-life adversity, recent stress, and neural development across different contexts and populations.

This study has several limitations. First, the measurement of childhood maltreatment relied on retrospective reports, which were identified as carrying inherent biases [58]. Ideally, future studies would benefit from utilizing prospective or objective assessments of childhood maltreatment to better determine how retrospective reporting biases might have affected the current results. Second, this sample was drawn from a UK-based cohort of healthy individuals aged 14 to 24; the findings may not be generalizable to other age groups or cultural settings. Hence, future research should seek to validate these results across more culturally diverse populations to examine their broader applicability. Expanding the age range of participants would also enhance the generalizability of these conclusions. Finally, while our study sheds light on the associations between childhood abuse, recent life stress, and resting-state network connectivity in healthy individuals, further research is warranted to corroborate our findings within a clinical trial. Such endeavors will provide insight into the neural mechanisms operating within clinical populations and facilitate the development of more targeted and effective interventions.

## 5. Conclusions

The findings of this study extend the stress sensitization model and deepen our understanding of the long-term effects of childhood abuse. Our findings indicate that childhood adversity, as a risk factor, may alter the relationship between current life stress and subsequent brain network connectivity. These insights offer valuable implications for clinical practitioners. It is crucial for clinicians to assess both the history of abuse and current stress levels in individuals with a background of childhood maltreatment, as these factors interact and significantly impact brain connectivity and mental health outcomes. Implementing regular neurodevelopmental monitoring alongside tailored psychological interventions can help mitigate the heightened sensitivity to stress observed in this population [59]. Furthermore, integrating neurofeedback [60,61] and mindfulness-based interventions [62] may support the stabilization of brain network connectivity. These strategies provide a comprehensive approach for clinicians to reduce the long-term impact of childhood abuse on mental health and neurodevelopment.

## Figures and Tables

**Table 1:** Sample characteristics.

Characteristic	*n* or Mean ± SD
No. of Subjects (Female)	172 (85)
Age	18.73 ± 2.80
Age group	
14–15	44
16–17	46
18–19	28
20–21	36
22–24	18
Ethnic	
White	148
Asian/Asian British	5
Mixed/multiple ethnic groups	14
Black/African/Caribbean/Black British	2
Other ethnic group	2
Decline to state	1
WASI	111.49 ± 10.04
Childhood abuse	18.15 ± 4.21
Childhood neglect	14.10 ± 4.49
Recent life stress	4.26 ± 1.93

**Table 2:** Longitudinal associations between recent life stress and network connectivity.

Network Connectivity at IUA2	*β*	95% CI	*p*	*p*FDR
within-ASN	0.110	[−0.044, 0.263]	0.160	0.477
within-CEN	0.086	[−0.067, 0.238]	0.267	0.518
within-DMN	−0.105	[−0.264, 0.053]	0.190	0.477
within-ERN	0.055	[−0.100, 0.209]	0.486	0.607
ASN-CEN	0.153	[0.005, 0.300]	0.043	0.430
ASN-DMN	0.073	[−0.084, 0.230]	0.363	0.518
ASN-ERN	0.078	[−0.074, 0.230]	0.311	0.518
CEN-DMN	0.015	[−0.145, 0.175]	0.852	0.852
CEN-ERN	0.119	[−0.030, 0.269]	0.117	0.477
DMN-ERN	−0.048	[−0.205, 0.110]	0.551	0.612

Note. Gender, age and network connectivity at IUA1 were incorporated as covariates throughout the analyses.

**Table 3:** The role of childhood abuse in moderating the relationship between recent life stress and network connectivity.

Network Connectivity at IUA2		Childhood Abuse × Recent Life Stress		
	*β*	95% CI	*p*	*p*FDR
within-ASN	0.209	[0.015, 0.403]	0.035	**0.050**
within-CEN	0.153	[−0.041, 0.347]	0.121	0.134
within-DMN	0.143	[−0.058, 0.345]	0.162	0.162
within-ERN	0.245	[0.054, 0.437]	0.012	**0.028**
ASN-CEN	0.253	[0.069, 0.437]	0.007	**0.025**
ASN-DMN	0.288	[0.093, 0.483]	0.004	**0.021**
ASN-ERN	0.280	[0.091, 0.469]	0.004	**0.021**
CEN-DMN	0.207	[0.004, 0.410]	0.046	0.057
CEN-ERN	0.236	[0.048, 0.423]	0.014	**0.028**
DMN-ERN	0.237	[0.040, 0.434]	0.018	**0.031**

Note. Gender, age, and network connectivity from IUA1 were accounted for as covariates in the analyses. Statistically significant *p* values, following FDR correction, are highlighted in bold.

## Data Availability

The data that support the findings of this study are available from the Neuroscience in Psychiatry Network, but restrictions apply to the availability of these data.

## Supplementary Materials

The following supporting information can be downloaded at https://www.mdpi.com/article/10.3390/bs14100958/s1. eMethods. Participant’s recruitment; eMethods. Image Preprocessing; Table S1. Regions of interest for the networks; Table S2. Moderating influences of childhood neglect on the association between recent life stress and network connectivity; Table S3. Correlation between participants’ gender, age, childhood abuse, childhood neglect and recent life stress; Table S4. Comparison of functional connectivity strength between age groups; Table S5. Comparison of functional connectivity strength between gender groups; Table S6. Moderating effects of childhood abuse on the association between recent life stress and network connectivity; Figure S1. Number of participants and reasons for incomplete enrollment. References [63,64,65,66,67,68] are cited in the supplementary materials.
