# Supplementary material for: Shedding Light on the Aftermath: Childhood Maltreatment’s Role in Modifying the Association Between Recent Life Stress and Resting-State Network Connectivity
Source: Behav Sci (Basel). 2024 Oct 16;14(10):958. doi: 10.3390/bs14100958 (PMC11505332; doi:10.3390/bs14100958)
Supplement: Supplementary file 1 [file behavsci-14-00958-s001.zip › behavsci-3204150-supplementary.pdf]

Supplementary Information for

Shedding light on the aftermath: Childhood Maltreatment's Role in Modifying the Association between Recent Life Stress and Resting-State Network Connectivity

This file include:

eMethods. Participant's recruitment

eMethods. Image Preprocessing

Table S1. Regions of interest for the networks

Table S2. Moderating influences of childhood neglect on the association between recent life stress and network connectivity

Table S3. Correlation between participants' gender, age, childhood abuse, childhood neglect and recent life stress

Table S4. Comparison of Functional Connectivity Strength Between Age Groups

Table S5. Comparison of Functional Connectivity Strength Between Gender Groups

Table S6. Moderating effects of childhood abuse on the association between recent life stress and network connectivity

Figure S1. Number of participants and reasons for incomplete enrollment.

eMethods. Participant's recruitment

Participants in this study were part of the NSPN cohort. The NSPN is a multi-center, accelerated longitudinal study aimed at measuring developmental changes in a demographically representative sample of 2,406 young people aged 14-24 years from North London and Cambridgeshire, UK (Kiddle et al., 2018). Participants were stratified by age and sex, with an equal number of males and females in each of the following age groups: 14-15, 16-17, 18-19, 20-21, and 22-25 years. The cohort's objective is to support an accelerated longitudinal design to measure developmental changes, which involves recruiting multiple, age-adjacent cohorts and following them longitudinally for a limited time period. This approach allows for a faster estimation of development across a wider range of ages than a single-cohort longitudinal follow-up.

Participants received a Home Questionnaire Pack (HQP) and a Sociodemographic Questionnaire to assess their mood, behavior, wellbeing, and demographic characteristics at three time-points (HQP1, HQP2, HQP3). Two in-unit assessments (IUA1, IUA2) comprised questionnaires, cognitive assessments, and MRI scanning. IUA1 and IUA2 interleaved with HQP1, HQP2 and HQP3. All evaluations were performed by trained research assistants under the supervision of licensed clinical psychologists. Detailed descriptions of the recruitment methods and sample are available in recent publications (Dorfschmidt et al., 2022; Vaghi et al., 2020; Váša et al., 2020).

A subsample of 318 healthy youth participated in an MRI study, with approximately 60 participants in each of five age bins (14 to 15 years, 16 to 17 years, 18 to 19 years, 20 to 21 years and 22 to 24 years). Participants were excluded if they reported a history of psychiatric treatment or neurological disorder, head injury, or intellectual disability. After rigorous visual quality control and excluding 10% of scans with highest during-scan motion, the final evaluable dataset included 298 participants. Of these, 281 subjects were scanned at baseline (IUA1) and 211 were scanned approximately 18 months later at follow-up (IUA2). For the present study, the final

sample included 172 participants (age range 14-25 years; mean [SD] age, 18.78 [2.85] years; 47.42% female) who were scanned at both time points. Further demographic information can be found in Table 1. When we employed a cut-off score of 17 or higher on the Mood and Feelings Questionnaire (MFQ) (Costello & Angold, 1988), as recommended by Yates et al. (2004), to identify mild-to-moderate depressive disorder in participants, we found that approximately 28% of participants at HPQ3 and approximately 30% of participants at HPQ2 who provided information on depression scored above this cut-off. This indicates a significant proportion of our sample experiencing depression. Participants aged 16-25 gave written informed consent for each aspect of the study; a legal guardian's written informed consent was obtained for those aged 14–15 years, and those youth gave assent to participate. The NSPN study was approved by the Cambridge Central Research Ethics Committee (12/EE/0250). This study was approved as a secondary data analysis protocol by the Institutional Review Board of our university.

eMethods. Image Preprocessing

Using Freesurfer v5.3.0, individual structural scans were processed with a pipeline including skull-stripping, segmentation of cortical grey and white matter, and reconstruction of the cortical surface and grey-white matter boundary (Fischl, Sereno, & Dale, 1999). Then all scans were precisely quality controlled by re-running the reconstruction algorithm after the addition of control points and white matter edits as previously described (Váša et al., 2020; Whitaker et al., 2016). The pre-processing of resting-state data for multiecho independent component analysis (ME-ICA) analysis was conducted in AFNI. Volumes obtained during steady-state equilibration (15 s) were disregarded. Matrices for deobliquing, motion correction, and anatomical-functional coregistration were computed, and subsequently combined into a single alignment matrix using the concatenation approach from the AFNI tool align_epi_anat.py. Slice timing correction, spatially aligned through application of the alignment matrix, and visual assessment for anatomical-functional coregistration were performed.

Furthermore, we employed ME-ICA during the pre-processing of functional scans to identify sources of variance in the fMRI time series that were BOLD-related and scaled linearly with TE, while discarding other sources of fMRI variance, such as head movement, which were not BOLD-related and did not scale with TE. In addition, we utilized realignment of scans to estimate six motion parameters (3 translation parameters and 3 rotation parameters) for each participant during pre-processing. These parameters were then used to calculate an overall estimate of motion, known as the framewise displacement (FD), which represents the sum of the absolute derivatives of the six motion parameters. To correct for head movement in each scan session, we used mean FD as a measure of head movement, and subsequently regressed functional connectivity on mean FD. This movement correction pipeline of ME-ICA followed by FD regression formed the basis for our analysis of functional connectivity. A broadband denoised fMRI time series at each voxel was generated by the retained independent components of the BOLD contrast. Finally, the BOLD signal oscillating in the frequency range 0.025-0.111 Hz was acquired after bandpass filtering using the discrete wavelet transform (Daubechies 4 wavelet). Detailed information about the pre-processing steps and ME- ICA can be found in published studies (Dorfschmidt et al., 2022; Vaghi et al., 2020; Váša et al., 2020).

Table S1. Regions of interest for the networks

Network	MNI Coordinates, x, y, z	Region
Anterior Salience Network	-6, 17, 47	L dorsal anterior cingulate cortex
	-31, 47, 22	L middle frontal gyrus
	-42, 14, -3	L anterior insula
	6, 17, 47	R dorsal anterior cingulate cortex
	28, 46, 26	R middle frontal gyrus
	-42, 14, -3	R anterior insula
Central Executive Network	-42, -63, 46	L inferior parietal lobule
	-32, 23, 49	L middle frontal gyrus
	-40, 48, -1	L middle frontal gyrus
	-59, -42, -12	L middle temporal gyrus
	-7, 34, 43	L medial frontal gyrus
	38, 26, 42	R middle frontal gyrus
	48, -54, 47	R inferior parietal lobule
	38, 54, 1	R middle frontal gyrus
	13, 2, 14	R caudate
	6, 37, 46	R medial frontal gyrus
Default Mode Network	-4, -52, 32	L posterior cingulate cortex
	-5, 55, -13	L ventromedial prefrontal cortex
	-49, -62, 34	L temporoparietal junction
	4, -53, 35	R posterior cingulate cortex
	5, 55, -13	R ventromedial prefrontal cortex
	50, -57, 36	R temporoparietal junction
Emotion Regulation Network	-6, 14, 58	L somatomotor area
	-42, 22, -6	L inferior frontal gyrus
	-44, 10, 46	L precentral gyrus
	-58, -38, -2	L middle temporal gyrus
	-42, -60, 44	L angular gyrus
	6, 14, 58	R somatomotor area
	50, 30, -8	R inferior frontal gyrus
	48, 8, 48	R precentral gyrus
	38, 22, 44	R middle temporal gyrus
	60, -54, 40	R angular gyrus

Table S2. Moderating influences of childhood neglect on the association between recent life stress and network connectivity

Network connectivity at IUA2	Childhood neglect ×recent life stress			
	β	95% CI	*p*	p_{FDR}
within-ASN	0.024	[-0.012, 0.060]	0.189	0.315
within-CEN	0.013	[-0.023, 0.049]	0.475	0.594
within-DMN	-0.005	[-0.042, 0.033]	0.806	0.806
within-ERN	0.027	[-0.009, 0.064]	0.137	0.273
ASN-CEN	0.034	[-0.001, 0.068]	0.053	0.266
ASN-DMN	0.029	[-0.007, 0.066]	0.117	0.273
ASN-ERN	0.038	[0.003, 0.073]	0.036	0.266
CEN-DMN	0.014	[-0.023, 0.052]	0.454	0.594
CEN-ERN	0.027	[-0.008, 0.062]	0.131	0.273
DMN-ERN	0.011	[-0.026, 0.049]	0.550	0.611

Note. Gender, age and network connectivity at IUA1 were included as covariates across the analyses.

Table S3. Correlation between participants' gender, age, childhood abuse, childhood neglect and recent life stress

Variables	Gender	Age	childhood abuse	childhood neglect	recent life stress
Gender	-				
Age	0.110	-			
childhood abuse	0.029	0.112	-		
childhood neglect	0.084	0.170*	0.631***	-	
recent life stress	0.104	0.195*	0.092	0.108	-

Note. *p < .05, **p < .01, ***p < .001

Table S4. Comparison of Functional Connectivity Strength Between Age Groups

Network connectivity at IUA2	Younger Age Group (Mean ± SD)	Older Age Group (Mean ± SD)	t	*p*	Effect Size (Cohen's d)
within-ASN	0.09±0.06	0.10±0.08	-0.817	0.414	-0.127
within-CEN	0.04±0.03	0.05±0.04	-1.413	0.159	-0.220
within-DMN	0.04±0.02	0.04±0.02	0.021	0.982	0.003
within-ERN	0.06±0.04	0.07±0.04	-1.666	0.099	-0.272
ASN-CEN	0.13±0.08	0.15±0.12	-0.884	0.378	-0.137
ASN-DMN	0.06±0.06	0.08±0.08	-1.678	0.095	-0.260
ASN-ERN	0.14±0.10	0.17±0.14	-1.483	0.140	-0.231
CEN-DMN	0.06±0.05	0.07±0.05	-1.105	0.270	-0.170
CEN-ERN	0.12±0.06	0.13±0.08	-1.215	0.225	-0.188
DMN-ERN	0.08±0.05	0.09±0.05	-1.234	0.218	-0.189

Table S5. Comparison of Functional Connectivity Strength Between Gender Groups

Network connectivity at IUA2	Male (Mean ± SD)	Female (Mean ± SD)	t	*p*	Effect Size (Cohen's d)
within-ASN	0.10±0.07	0.09±0.06	-1.488	0.138	0.226
within-CEN	0.05±0.03	0.04±0.03	-1.153	0.250	0.175
within-DMN	0.04±0.02	0.04±0.02	0.712	0.477	-0.108
within-ERN	0.08±0.05	0.07±0.03	-1.838	0.063	0.288
ASN-CEN	0.15±0.10	0.13±0.09	-1.092	0.275	0.166
ASN-DMN	0.08±0.08	0.06±0.07	-1.412	0.159	0.215
ASN-ERN	0.17±0.14	0.14±0.09	-1.766	0.077	0.274
CEN-DMN	0.06±0.05	0.06±0.05	-0.718	0.473	0.109
CEN-ERN	0.13±0.07	0.11±.07	-1.723	0.086	0.262
DMN-ERN	0.09±0.05	0.08±0.05	-1.503	0.134	0.229

Table S6. Moderating effects of childhood abuse on the association between recent life stress and network connectivity

Network connectivity at IUA2	Childhood abuse × recent life stress			
	β	95% CI	p	p_{FDR}
within-ASN	0.210	[0.017, 0.403]	0.034	**0.049**
within-CEN	0.154	[-0.039, 0.347]	0.120	0.122
within-DMN	0.145	[-0.056, 0.347]	0.159	0.160
within-ERN	0.243	[0.052, 0.434]	0.013	**0.029**
ASN-CEN	0.252	[0.069, 0.436]	0.007	**0.025**
ASN-DMN	0.293	[0.100, 0.485]	0.003	**0.020**
ASN-ERN	0.281	[0.093, 0.469]	0.004	**0.020**
CEN-DMN	0.209	[0.007, 0.411]	0.043	0.055
CEN-ERN	0.236	[0.048, 0.423]	0.014	**0.028**
DMN-ERN	0.239	[0.043, 0.435]	0.018	**0.030**

Note. Gender, age, SES, education level and network connectivity at IUA1 were included as covariates across the analyses. *P* values after FDR correction are highlighted in bold to emphasize their statistical significance.

Figure S1. Number of participants and reasons for incomplete enrollment.

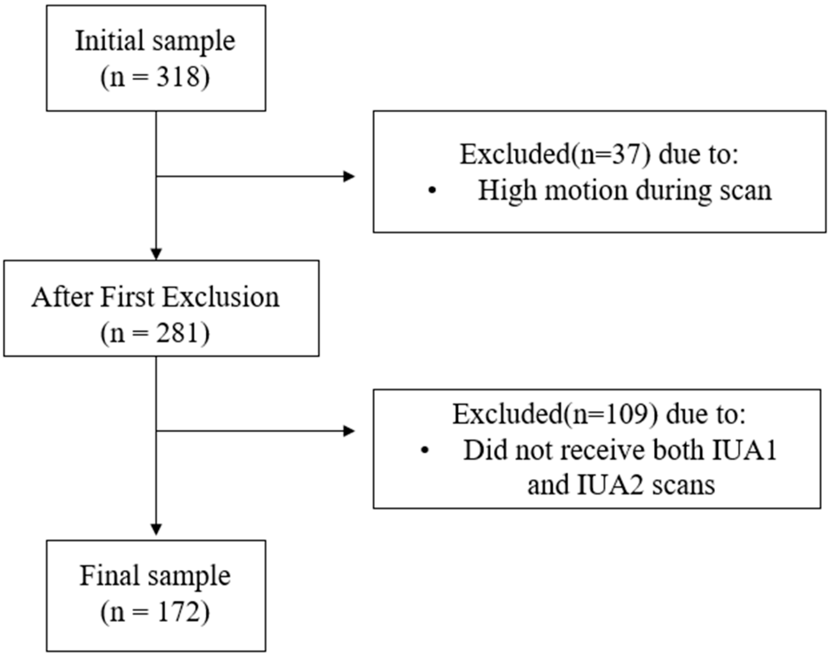

References

Costello, E. J., & Angold, A. (1988). Scales to assess child and adolescent depression: checklists, screens, and nets. *Journal of the American Academy of Child and Adolescent Psychiatry, 27*(6), 726-737. doi:10.1097/00004583-198811000-00011

Dorfschmidt, L., Bethlehem, R. A., Seidlitz, J., Váša, F., White, S. R., Romero-García, R., . . . Goodyer, I. M. (2022). Sexually divergent development of depression-related brain networks during healthy human adolescence. *Science Advances, 8*(21), eabm7825.

Fischl, B., Sereno, M. I., & Dale, A. M. (1999). Cortical surface-based analysis: II: inflation, flattening, and a surface-based coordinate system. *Neuroimage, 9*(2), 195-207.

Vaghi, M. M., Moutoussis, M., Váša, F., Kievit, R. A., Hauser, T. U., Vértes, P. E., . . . Bullmore, E. T. (2020). Compulsivity is linked to reduced adolescent development of goal-directed control and frontostriatal functional connectivity. *Proceedings of the National Academy of Sciences, 117*(41), 25911-25922.

Váša, F., Romero-Garcia, R., Kitzbichler, M. G., Seidlitz, J., Whitaker, K. J., Vaghi, M. M., . . . Dolan, R. J. (2020). Conservative and disruptive modes of adolescent change in human brain functional connectivity. *Proceedings of the National Academy of Sciences, 117*(6), 3248-3253.

Whitaker, K. J., Vértes, P. E., Romero-Garcia, R., Váša, F., Moutoussis, M., Prabhu, G., . . . Rittman, T. (2016). Adolescence is associated with genomically patterned consolidation of the hubs of the human brain connectome. *Proceedings of the National Academy of Sciences, 113*(32), 9105-9110.
